# Autoinducer 2 Signaling via the Phosphotransferase FruA Drives Galactose Utilization by *Streptococcus pneumoniae*, Resulting in Hypervirulence

**DOI:** 10.1128/mBio.02269-16

**Published:** 2017-01-24

**Authors:** Claudia Trappetti, Lauren J. McAllister, Austen Chen, Hui Wang, Adrienne W. Paton, Marco R. Oggioni, Christopher A. McDevitt, James C. Paton

**Affiliations:** a Department of Molecular and Cellular Biology, Research Centre for Infectious Diseases, University of Adelaide, Adelaide, Australia; b Department of Genetics, University of Leicester, Leicester, United Kingdom; University of Mississippi Medical Center

## Abstract

Communication between bacterial cells is crucial for the coordination of diverse cellular processes that facilitate environmental adaptation and, in the case of pathogenic species, virulence. This is achieved by the secretion and detection of small signaling molecules called autoinducers, a process termed quorum sensing. To date, the only signaling molecule recognized by both Gram-positive and Gram-negative bacteria is autoinducer 2 (AI-2), synthesized by the metabolic enzyme LuxS (*S*-ribosylhomocysteine lyase) as a by-product of the activated methyl cycle. Homologues of LuxS are ubiquitous in bacteria, suggesting a key role in interspecies, as well as intraspecies, communication. Gram-negative bacteria sense and respond to AI-2 via the Lsr ABC transporter system or by the LuxP/LuxQ phosphorelay system. However, homologues of these systems are absent from Gram-positive bacteria and the AI-2 receptor is unknown. Here we show that in the major human pathogen *Streptococcus pneumoniae*, sensing of exogenous AI-2 is dependent on FruA, a fructose-specific phosphoenolpyruvate-phosphotransferase system that is highly conserved in Gram-positive pathogens. Importantly, AI-2 signaling via FruA enables the bacterium to utilize galactose as a carbon source and upregulates the Leloir pathway, thereby leading to increased production of capsular polysaccharide and a hypervirulent phenotype.

## IMPORTANCE

*S. pneumoniae* is a Gram-positive bacterium frequently carried asymptomatically in the human nasopharynx. However, in a proportion of cases, it can spread to other sites of the body, causing life-threatening diseases that translate into massive global morbidity and mortality. Our data show that AI-2 signaling via FruA promotes the transition of the pneumococcus from colonization to invasion by facilitating the utilization of galactose, the principal sugar available in the upper respiratory tract. AI-2-mediated upregulation of Leloir pathway enzymes results in increased production of capsular polysaccharide and hypervirulence in a murine intranasal challenge model. This identifies the highly conserved FruA phosphotransferase system as a target for new antimicrobials based on the disruption of this generic quorum-sensing system.

## INTRODUCTION

*Streptococcus pneumoniae* (the pneumococcus) is one of the world’s foremost bacterial pathogens, killing 1 to 2 million people each year. In spite of this, it is considered part of the “normal” nasopharyngeal microflora, asymptomatically colonizing up to 95% of individuals. In a proportion of such carriers, pneumococci from this mucosal beachhead can penetrate deeper tissues, causing a variety of diseases, including pneumonia, bacteremia, meningitis, and otitis media, which collectively inflict massive global morbidity and mortality (1–3).

Although *S. pneumoniae* is probably one of the most studied bacterial pathogens, the mechanism behind its transition from colonizer to pathogen remains a mystery. The capacity to form a biofilm on host mucosal surfaces is increasingly being recognized as a critical event in the pathogenesis of pneumococcal disease (4). Within these communities, bacteria exchange both metabolic signals and metabolizable factors, but they can also communicate via a series of small diffusible autoinducer (AI) molecules that specifically induce changes in gene expression in target cells in a density-dependent fashion. Such interactions are grouped under the general term quorum-sensing (QS) systems (5). QS systems enable bacteria to monitor their population density by releasing AIs and subsequently responding to threshold concentrations of accumulated AIs.

QS systems can be divided into three major classes: (i) LuxI/LuxR-type QS in Gram-negative bacteria, (ii) oligopeptide two-component QS in Gram-positive bacteria, and (iii) LuxS-mediated AI-2 QS in both Gram-positive and Gram-negative bacteria (6–9). The LuxS/AI-2 QS system is believed to function in interspecies communication in polymicrobial communities, such as in the human nasopharynx. The synthesis of AI-2 is catalyzed by the metabolic enzyme LuxS, which generates the precursor compound 4,5-dihydroxy-2,3-pentanedione (DPD) as a by-product of the conversion of *S*-ribosylhomocysteine into homocysteine, an integral reaction of the activated methyl cycle (10). Gram-negative bacteria such as *Escherichia coli* and *Salmonella* spp. sense and respond to AI-2 via the Lsr ABC transporter system, while in *Vibrio* spp., a furanosyl-borate-diester form of AI-2 is detected by the LuxP/LuxQ phosphorelay system (11). However, homologues of these systems are absent from Gram-positive bacteria and the AI-2 receptor is unknown.

DPD is essentially a pentose with two keto groups. Low-G+C Gram-positive bacteria, such as *S. pneumoniae*, are characterized by having a large array of carbohydrate binding and transport proteins on their surface (12). These proteins are the prime candidates for the binding of any extracellular sugars and are therefore also the likely transporters for ketopentose sugars such as AI-2. *S. pneumoniae* has 30 sugar uptake systems, including 21 phosphotransferase systems (PTS). These systems use phosphoenolpyruvate as an energy source for the uptake of their substrates and as a phosphoryl donor for their phosphorylation. The use of a PTS to transport AI-2 in pneumococci is an attractive hypothesis, given that the incoming QS molecule would be phosphorylated, a modification that is known to occur in Lsr-mediated AI-2 uptake in Gram-negative species (13, 14). Phosphorylated AI-2 would be unable to cross the cell membrane, enabling intracellular accumulation of AI-2-P in accordance with the extracellular AI-2 concentration, thereby enabling a cell density-dependent response.

A previous study has suggested that the LuxS/AI-2 QS system is directly involved in disease pathogenesis in many bacterial species (11). In a mouse intranasal (i.n.) challenge model, an *S. pneumoniae luxS* mutant was less able than wild-type strain D39 to spread from the nasopharynx to the lungs or blood (15). The mutant also showed defects in several *in vitro* virulence-related phenotypes, including the capacity to form a biofilm, iron uptake, and genetic competence (16). However, such studies with *luxS* mutants alone cannot discriminate phenotypes dependent on AI-2 signaling from general metabolic effects of interruption of the activated methyl cycle. In the present study, we showed that exogenous AI-2 could complement defects in virulence-related *in vitro* and *in vivo* phenotypes of *luxS* mutants and also identified the mechanism whereby AI-2 modulates the virulence of *S. pneumoniae*.

## RESULTS

### Exogenous AI-2 complements *luxS* mutant growth phenotypes.

**FIG 1:**
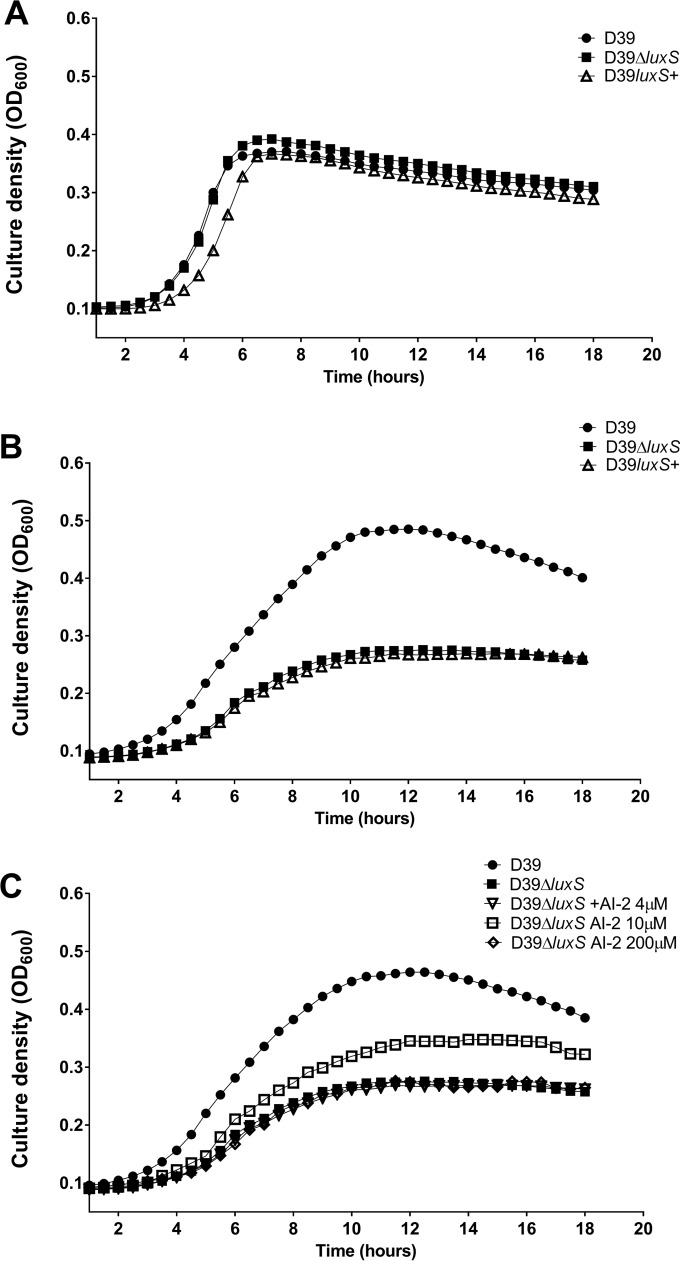
Impact of AI-2 on bacterial growth. D39, D39Δ*luxS*, and D39*luxS*^+^ were cultured in C+Y-Glc (A) or C+Y-Gal (B), and growth was monitored by measuring the OD_600_. (C) The growth of D39 and D39Δ*luxS* in C+Y-Gal supplemented with 0, 4, 10, or 200 μM AI-2 was monitored. Data are mean values of triplicate cultures.

The primary habitat of *S. pneumoniae* is the human upper respiratory tract, where the principal sugar available for use as a carbon source is galactose (17, 18). However, previous studies of the growth properties of *S. pneumoniae luxS* mutants have employed media that contain glucose, and no growth defects were reported (16, 19, 20). We therefore commenced by comparing the growth phenotypes of serotype 2 *S. pneumoniae* strain D39 with that of its *luxS* deletion mutant (D39Δ*luxS*) and a *luxS*-overexpressing strain (D39*luxS*^+^) in C+Y medium (21) containing either glucose (C+Y-Glc) (Fig. 1A) or galactose (C+Y-Gal) (Fig. 1B). In C+Y-Glc, the growth kinetics of D39 and D39Δ*luxS* were virtually identical, while there was a slight growth retardation of the D39*luxS*^+^ strain (Fig. 1A). In contrast, in C+Y-Gal, there was a marked increase in the generation time and a reduction in the final cell density of both D39Δ*luxS* and D39*luxS*^+^ compared to those of the D39 parent strain (Fig. 1B). This suggested that either too little or too much *luxS* expression may be detrimental in this medium. To distinguish between true QS effects mediated by AI-2 from the indirect consequences of *luxS* disruption on the activated methyl cycle, we then examined the capacity of various concentrations of exogenous purified DPD (AI-2) to complement the growth defect of D39Δ*luxS* in C+Y-Gal. Supplementation of C+Y-Gal with 10 μM AI-2 partially restored growth, but supplementation with lower (4 μM) or higher (200 μM) AI-2 concentrations had no impact whatsoever (Fig. 1C). Furthermore, the growth defect of D39*luxS*^+^ in C+Y-Gal could not be complemented by any of these concentrations of AI-2 (result not presented). Taken together, these findings indicate that extracellular AI-2 is either sensed or imported into the cell and that this process appears to be under carbon catabolite repression.

### Exogenous AI-2 increases *S. pneumoniae* virulence.

**FIG 2:**
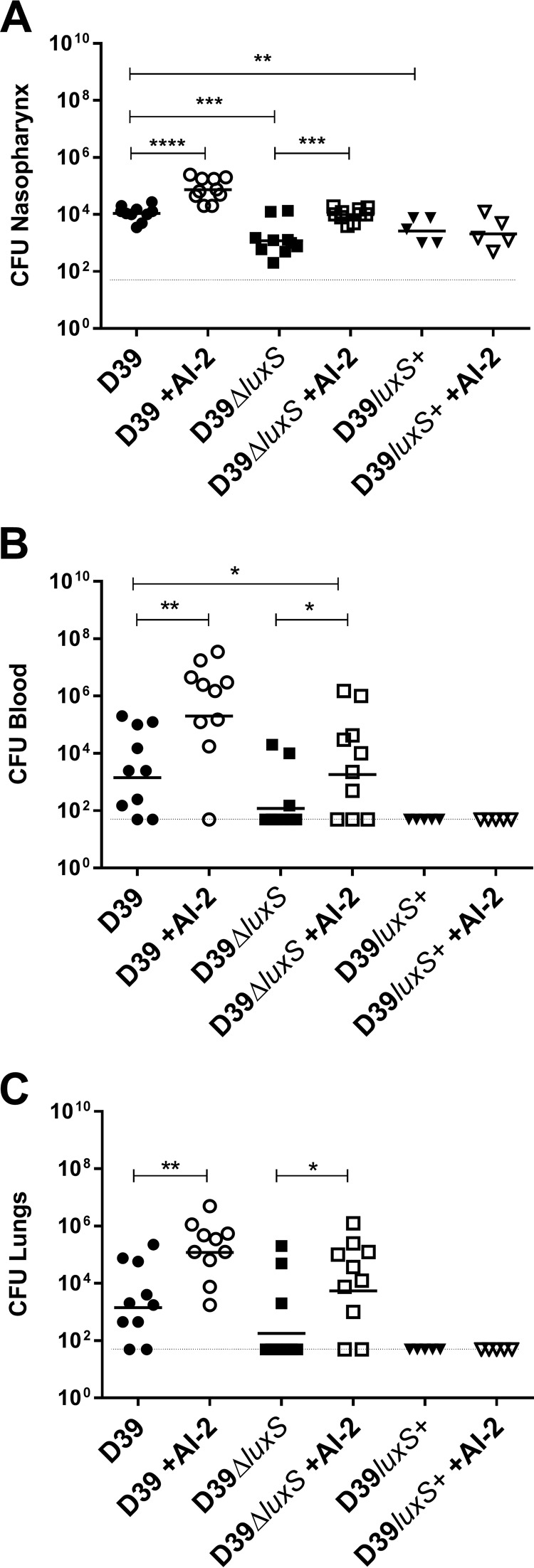
Exogenous AI-2 increases virulence of *S. pneumoniae*. Mice were challenged i.n. with 10^7^ CFU of D39, D39Δ*luxS*, or D39*luxS*^+^ with i.n. administration of 10 μl of PBS with or without 1 μg of AI-2 at 0, 6, 12, and 18 h postchallenge. Bacterial loads in the nasopharynx (A), blood (B), and lungs (C) were determined at 24 h. Log-transformed data were analyzed by Student’s unpaired (two-tailed) *t* test: *, *P* < 0.05; **, *P* < 0.01; ***, *P* < 0.001; ****, *P* < 0.0001.

We then determined whether the known virulence defect of a *luxS* mutant relative to its wild-type parent D39 (15) could be complemented by administration of exogenous synthetic AI-2. Mice were challenged i.n. with *S. pneumoniae* D39, D39Δ*luxS*, or D39*luxS*^+^ with or without AI-2 administered i.n. at time zero and at 6, 12, and 18 h postinfection. Mice challenged with D39Δ*luxS* had significantly lighter bacterial loads in the nasopharynx, lungs, and blood at 24 h postchallenge than those challenged with D39 (Fig. 2). However, i.n. administration of AI-2 significantly increased the bacterial loads in all host niches, such that the virulence of D39Δ*luxS* with AI-2 was indistinguishable from that of D39 without AI-2, while D39 with AI-2 became hypervirulent. In contrast, mice challenged with D39*luxS*^+^ had significantly lighter bacterial loads in the nasopharynx than D39-challenged mice (Fig. 2A) and no bacteria could be detected in the lungs or blood of these mice. Moreover, unlike the findings with D39- and D39Δ*luxS*-infected mice, i.n. administration of AI-2 did not increase the numbers of D39*luxS*^+^ bacteria in any of the host niches (Fig. 2A to C).

**FIG 3:**
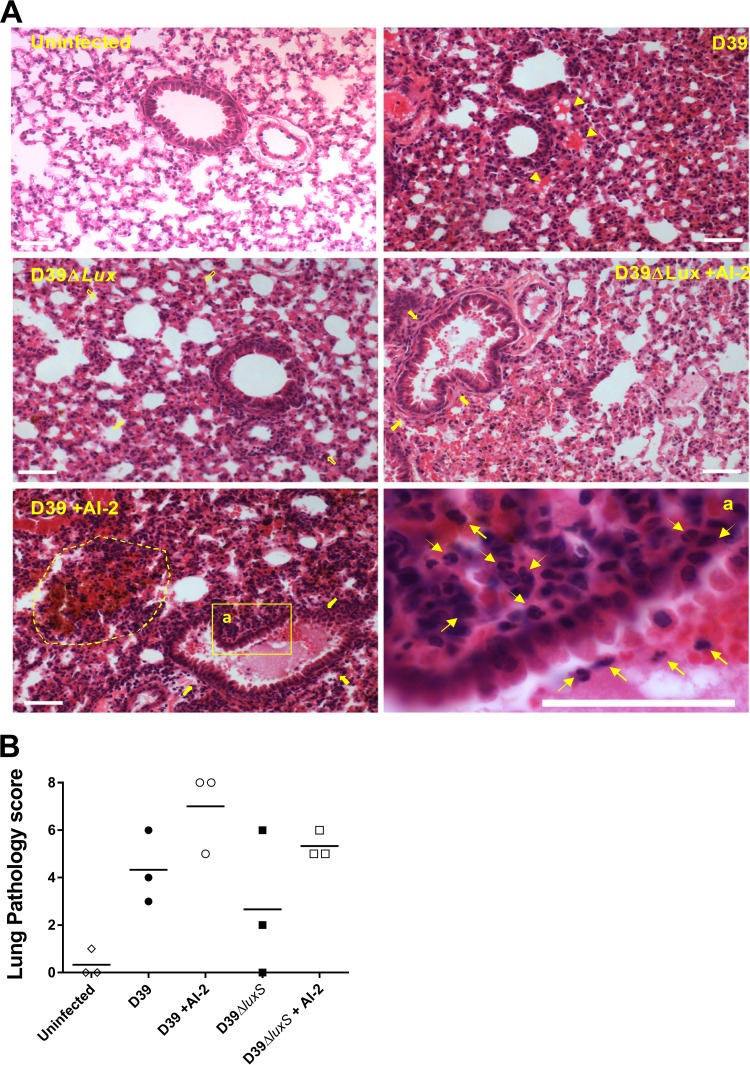
Histological examination. Mice were challenged i.n. with D39 or D39Δ*luxS* with or without AI-2 treatment at 0 and 6 h. Lung sections from mice at 12 h after the i.n. challenge were stained with H&E and examined by light microscopy (A) (scale bars are 0.05 mm). Lung sections were also scored according the following scheme: congested capillaries (arrowheads), scored 0 to 2; thickened alveolar wall (hollow arrows), scored 0 to 2; secretion and hemorrhage in the alveolar and bronchiolar spaces (solid arrows), scored 0 to 2; parenchymal hemorrhage (dotted line) and neutrophil infiltration (arrows), scored 0 to 2. The total pathological score (maximum = 8) is shown for each mouse (*n* = 3 per group) (B). A horizontal bar denotes the mean value of each group.

Histological analysis of the infected lungs at 12 h postinfection reflected the above findings (Fig. 3A). Lungs from mice challenged with D39Δ*luxS* exhibited less severe inflammation than those from mice challenged with D39, as evidenced by hematoxylin-and-eosin (H&E)-stained sections, resulting in a lower mean pathological score (Fig. 3B). Two out of three mice challenged with D39Δ*luxS* showed no or only mild inflammation signs in the lung tissue (only a minor degree of swelling of alveolar/bronchiole walls and minor lung tissue leukocyte infiltration), similar to those of uninfected mice (Fig. 3A, D39Δ*luxS* panel). Importantly, AI-2 treatment of both D39- and D39Δ*luxS*-infected mice increased the degree of inflammation, such that the lungs of mice challenged with D39Δ*luxS* and treated with AI-2 were similar to those of mice challenged with D39, while the degree of inflammation in the lungs of mice challenged with D39 and treated with synthetic AI-2 was higher than that of mice challenged with D39. AI-2 treatment increased the mean pathological score of lungs challenged with D39Δ*luxS* to 5.0, compared with 4.3 for those challenged with D39, while AI-2 treatment of D39-challenged mice increased the mean pathological score to 7.0 (Fig. 3B).

As further confirmation that exogenous AI-2 increases virulence, mice (*n* = 5 per group) challenged with D39 and treated with AI-2 as described above had a median survival time of 53 h, compared to 73 h for those given phosphate-buffered saline (PBS) (*P* < 0.05; Mann-Whitney U test). Furthermore, at 48 h, the geometric mean bacterial loads in the blood were 5.83 × 10^8^ and 7.53 × 10^6^ CFU/ml, respectively (*P* < 0.01).

Thus, we conclude that the virulence defect of D39Δ*luxS* is attributable to loss of AI-2 signaling rather than any metabolic side effect of perturbation of the activated methyl cycle and that exogenous AI-2 directly increases virulence. The nonvirulent phenotype observed in D39*luxS*^+^ and the lack of *in vivo* efficacy of exogenous AI-2 in this strain are consistent with the *in vitro* growth defect observed in C+Y-Gal, as well as the inability of exogenous AI-2 to restore growth (Fig. 1C).

**FIG 4:**
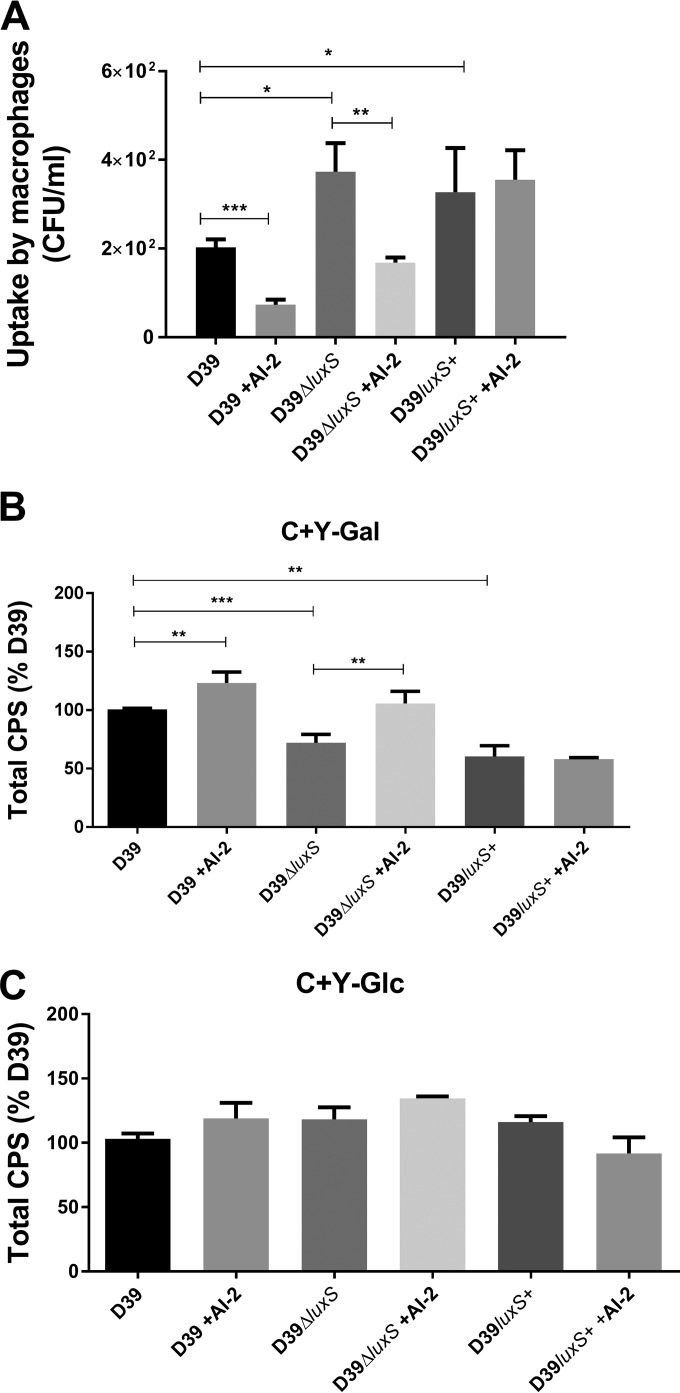
Uptake by macrophages and total CPS production. (A) D39, D39Δ*luxS*, and D39*luxS*^+^ were incubated with differentiated THP-1 cells after growth in C+Y-Gal with or without 10 μM AI-2. Extracellular bacteria were killed by penicillin and gentamicin treatment, and internalized pneumococci were enumerated. Total CPS production by D39, D39Δ*luxS*, and D39*luxS*^+^ grown to an OD_600_ of 0.2 in C+Y-Gal (B) or C+Y-Glc (C) with or without 10 μM AI-2 was determined by uronic acid assay. Data are the mean ± the standard deviation of triplicate assays and are expressed as a percentage of that for D39 without AI-2. Data were analyzed by Student’s unpaired (two-tailed) *t* test: *, *P* < 0.05; **, *P* < 0.01; ***, *P* < 0.001.

Bacterial loads in a given tissue type are determined not only by the growth rate but also by the rate of clearance by innate immune cells. We therefore examined the capacity of THP-1-derived human macrophages to internalize the various strains with or without exogenous AI-2. The numbers of intracellular bacteria in macrophages infected with D39Δ*luxS* and D39*luxS*^+^ were significantly greater than (roughly double) that of wild-type D39-infected cells (Fig. 4A). Preincubation of the bacteria with synthetic AI-2 significantly decreased the ability of the macrophages to internalize D39 or D39Δ*luxS*, but there was no effect on the uptake of D39*luxS*^+^ (Fig. 4A). Thus, exogenous AI-2 significantly increases the resistance of pneumococci to internalization by macrophages.

### AI-2 promotes capsule production.

In view of these findings, we hypothesized that the stimulatory effects of exogenous AI-2 on virulence of *S. pneumoniae* D39, and the attenuating effect of *luxS* mutation, could be due to opposite impacts on capsular polysaccharide (CPS) production, as the capsule is known to be strongly antiphagocytic (22). Accordingly, we measured the total amount of type 2 CPS in cells grown in C+Y-Glc or C+Y-Gal. Production of type 2 CPS was significantly lower in the D39Δ*luxS* and D39*luxS*^+^ strains than in wild-type D39 in C+Y-Gal (Fig. 4A), which is consistent with the attenuated virulence of these mutants. Moreover, exogenous AI-2 restored CPS production by D39Δ*luxS* to a level commensurate with that of the wild type and induced a significant increase in CPS production by D39 (Fig. 4B), but again, AI-2 had no effect on D39*luxS*^+^. Thus, CPS production *in vitro* paralleled the hypervirulence of D39 in the presence of exogenous AI-2 *in vivo*. However, when cells were grown in C+Y-Glc, there were no significant differences in either bacterial growth or CPS production among D39Δ*luxS*, D39*luxS*^+^, and wild-type D39, and exogenous AI-2 had no effect whatsoever (Fig. 4C). Moreover, the total CPS production by D39 in C+Y-Glc was less than half of that seen in cells grown in C+Y-Gal (result not shown), consistent with previous observations (23).

### Global gene expression analysis of AI-2-treated cells.

**FIG 5:**
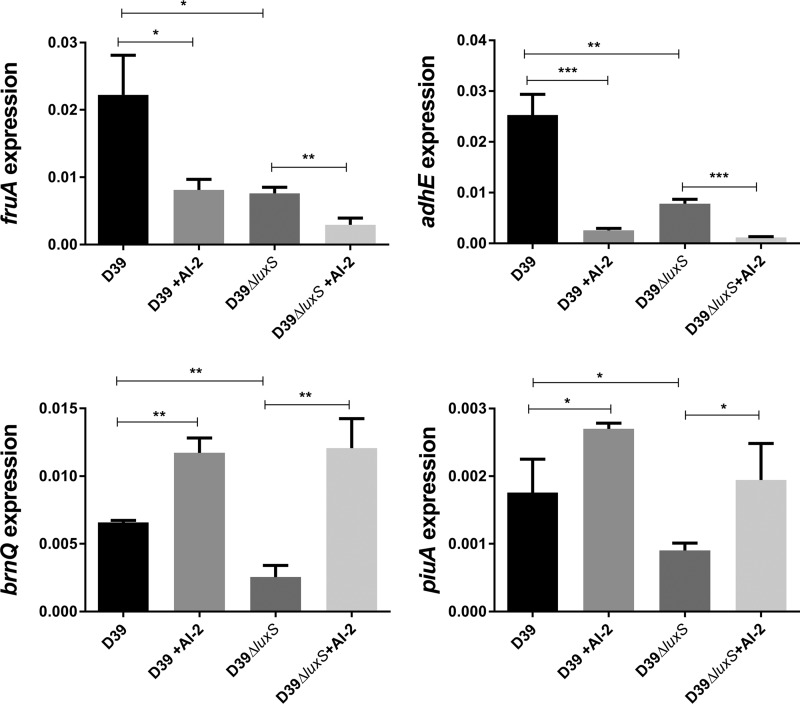
Relative expression of *fruA*, *adhE*, *spd_0546*, and *piuA* by D39 and D39Δ*luxS* grown in C+Y with or without 10 μM AI-2 was quantitated by qRT-PCR with 16S rRNA as an internal standard. Data are total expression relative to 16S rRNA (mean ± the standard deviation of three independent experiments). *, *P* < 0.05; **, *P* < 0.01; ***, *P* < 0.001 (Student’s unpaired *t* test).

We then performed a transcriptomic analysis to examine the global effect of exogenous AI-2 on gene expression patterns in D39 and D39Δ*luxS* (Table 1). For these studies, cells were grown in C+Y without added sugars and RNA was extracted 30 min after the addition of 10 μM AI-2. Addition of AI-2 caused significant changes in the expression of 22 genes in D39 but only 9 genes in D39Δ*luxS* (adjusted *P* < 0.05). Importantly, no differences in the expression of any of the genes in the CPS biosynthesis (*cps*) locus were observed in either strain. These data indicate that the impact of AI-2 on total CPS production, and hence virulence, is independent of *cps* transcription. However, AI-2 did induce significant differences in the expression of four genes/operons in both strains, notably, an operon (*spd_0771-0773*) comprising *lacR1*, *fruB*, and *fruA* (encoding the lactose phosphotransferase system repressor, phosphofructokinase, and fructose PTS enzyme IIABC, respectively), *piuA* (iron-binding lipoprotein), *adhE* (an iron-containing aldehyde-alcohol dehydrogenase), and *brnQ* (branched-chain amino acid transport system II carrier) (Table 1). The differential expression of these genes was confirmed by real-time quantitative reverse transcription (qRT)-PCR analysis (Fig. 5). Interestingly, in both D39 and D39Δ*luxS*, *fruA* and *adhE* were repressed in the presence of AI-2 whereas *brnQ* and *piuA* were induced. However, the transcription of all four genes was lower in D39Δ*luxS* than in D39.

**TABLE 1:** Differential gene expression in D39 relative to that in D39Δ*luxS* with or without AI-2^a^

D39 ORF^b^	Gene	Description	D39 vs D39Δ*luxS*	D39Δ*luxS* vs D39Δ*luxS* + AI-2	D39 vs D39 + AI-2
SPD_0091		Conserved hypothetical protein		2.7	1.7
SPD_0114		Hypothetical protein	3.9		
SPD_0115		Hypothetical protein	3.9		
SPD_0116		Hypothetical protein	4.5		
SPD_0117		Hypothetical protein	0.4		
SPD_0265	*adhA*	Alcohol dehydrogenase			0.7
SPD_0309	*luxS*	LuxS *S*-ribosylhomocysteine lyase	0.0003		
SPD_0334	*aliA*	Oligopeptide-binding protein AliA	2.1		
SPD_0404	*ilvB*	Lactose phosphotransferase system repressor	1.7		
SPD_0405	*ilvN*	Acetolactate synthase	1.7		
SPD_0406	*ilvC*	Ketol-acid reductoisomerase	1.7		
SPD_0407		Conserved hypothetical protein	1.8		
SPD_0408		Conserved hypothetical protein	1.8		
SPD_0420	*pflB*	Formate acetyltransferase		0.4	0.6
SPD_0451		Type I restriction-modification system	1.7		
**SPD_0546**	***brnQ***	**Branched-chain amino acid transport system II** **carrier protein**	**1.5**	**2.9**	**2.3**
SPD_0558	*prtA*	Cell wall-associated serine protease PrtA		0.5	
SPD_0732		30S ribosomal protein			1.6
**SPD_0771**	***lacR1***	**Lactose phosphotransferase system repressor**	**1.9**	**0.3**	**0.6**
**SPD_0772**	***fruB***	**1-Phosphofructokinase**	**1.9**	**0.3**	**0.5**
**SPD_0773**	***fruA***	**PTS, fructose-specific IIABC components**	**1.7**	**0.4**	0.7^c^
SPD_0900	*asd*	Aspartate-semialdehyde dehydrogenase	1.6		
SPD_0901	*dapA*	Dihydrodipicolinate synthase	1.6		
SDP_1004	*gapN*	Glyceraldehyde-3-phosphate dehydrogenase			0.6
SPD_1256		Peptidase, U32 family protein			0.6
SPD_1257		Conserved hypothetical protein			0.6
SPD_1258		Peptidase, U32 family protein			0.6
SPD_1360		Conserved hypothetical protein			1.7
SPD_1408		Conserved hypothetical protein	1.8		1.7
SPD_1415		Oxidoreductase, pyridine nucleotide-disulfide			1.5
SPD_1504	*nanA*	Sialidase A (neuraminidase A)			0.5
SPD_1563	*dctA*	Serine/threonine transporter SstT	1.6		
**SPD_1649**	***piuA***	**Iron compound ABC transporter**	**1.6**	**2.0**	**1.6**
SPD_1650		Iron compound ABC transporter			1.5
**SPD_1834**	***adhE***	**Bifunctional acetaldehyde-coenzyme A/alcohol** **dehydrogenase**	**2.0**	**0.1**	**0.1**
SPD_1956	*ilvD*	Dihydroxy-acid dehydratase			0.6
SPD_2012	*glpO*	Alpha-glycerophosphate oxidase			1.7
SPD_2013	*glpK*	Glycerol kinase			1.6

a Differential gene expression was determined by RNA-seq analysis as described in Materials and Methods. Data shown are fold changes in gene expression for the comparisons indicated. Genes commonly differentially expressed in all three comparisons are shown in bold type. Only genes with statistically significant differential expression (adjusted *P* < 0.05) are shown, except where indicated otherwise.

b ORF, open reading frame.

c No statistically significant differential expression.

**FIG 6:**
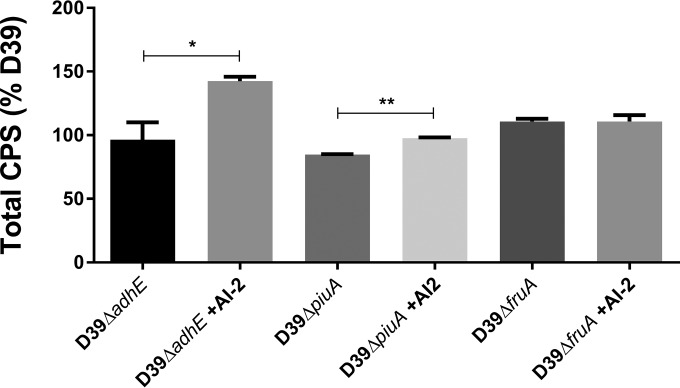
CPS production by D39Δ*adhE*, D39Δ*piuA*, and D39Δ*fruA* after growth in C+Y-Gal to an OD_600_ of 0.2 with or without 10 μM AI-2 was determined by uronic acid assay. Data are the mean ± the standard deviation of triplicate assays and are expressed as a percentage of D39 without AI-2. *, *P* < 0.05; **, *P* < 0.01 (Student’s unpaired *t* test).

We therefore constructed D39 derivatives with deletion mutations in the four AI-2-regulated genes identified above to determine whether any play a direct role in AI-2 sensing by *S. pneumoniae*. The *fruA* gene was a particularly promising candidate, as it encodes a fructose-PTS uptake system component that might be capable of recognizing a ketopentose like AI-2. Moreover, PTS uptake systems typically phosphorylate their cargo, resulting in intracellular retention and accumulation, a critical feature of cell density-dependent signaling molecules such as AI-2 (12). In the first instance, we used CPS production in C+Y-Gal as a readout of AI-2-mediated signaling. Similar to the earlier observations for D39 and D39Δ*luxS*, exogenous AI-2 increased the total CPS in D39Δ*piuA* and D39Δ*adhE*. Strikingly, AI-2 had no impact whatsoever on CPS production by D39Δ*fruA* (Fig. 6). Moreover, when tested in the murine i.n. challenge model (*n* = 5 per group), D39Δ*fruA* was at least as virulent as D39, but importantly, treatment with exogenous AI-2 had no effect on the loads of D39Δ*fruA* bacteria in the nasopharynx, blood, and lungs at 24 h postchallenge. Respective geometric mean bacterial loads in the absence or presence of AI-2 were 7.94 × 10^3^ versus 1.10 × 10^4^ CFU in the nasopharynx, 4.07 × 10^5^ versus 1.55 × 10^5^ CFU/ml in the blood, and 7.11 × 10^5^ versus 1.20 × 10^5^ CFU in the lungs. However, the impact of the *brnQ* deletion on CPS production could not be assessed in the D39Δ*brnQ* strain because of a major *in vitro* growth defect.

**FIG 7:**
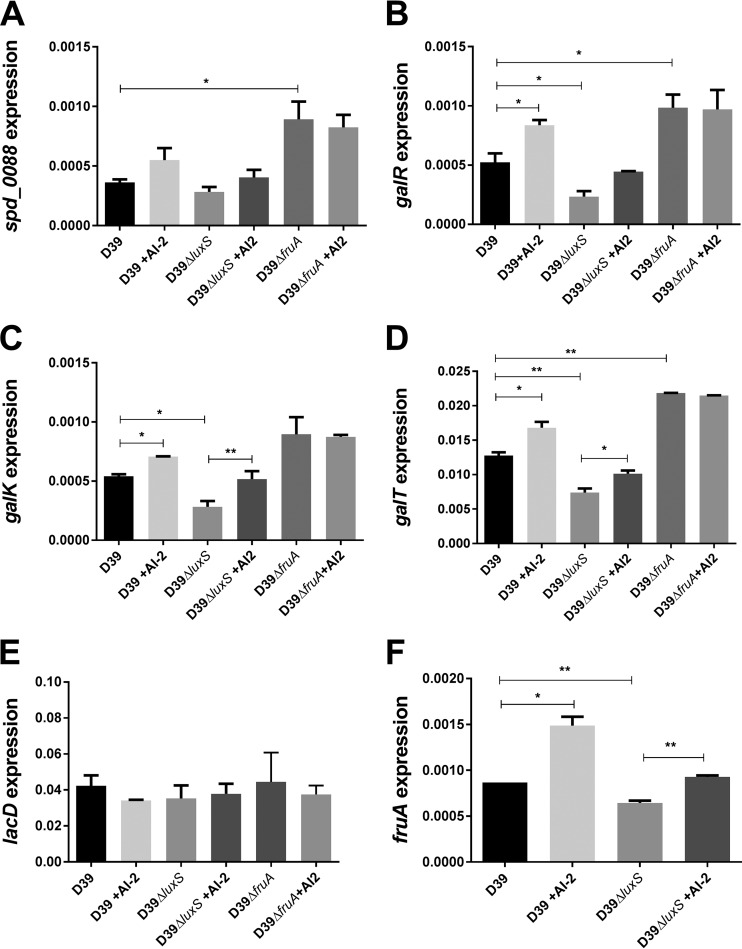
Relative expression of *spd_0088* (A), *galR* (B), *galK* (C), *galT* (D), *lacD* (E), and *fruA* (F) by D39, D39Δ*luxS*, and D39Δ*fruA* grown in CDM-Gal with or without 10 μM AI-2 was quantitated by qRT-PCR with 16S rRNA as an internal standard as described in Materials and Methods. Data are the total expression relative to 16S rRNA (mean ± the standard deviation from three independent experiments). *, *P* < 0.05; **, *P* < 0.01 (Student’s unpaired *t* test).

Collectively, the above findings indicate that AI-2 signaling in *S. pneumoniae* is dependent on FruA and suggest that the impacts on growth, CPS production, and virulence may be mediated via effects on Gal uptake and/or catabolism. In *S. pneumoniae*, there are two pathways for Gal uptake and utilization: a PTS (SPD_0559-0561) whereby Gal-6-phosphate (Gal6P) is fed into the glycolytic pathway via the tagatose-6-phosphate pathway and a Gal-specific ABC transporter (SPD_0088-0090) that delivers free Gal into the Leloir pathway. The latter pathway is critically important, as it generates Glc-1-phosphate (G1P), from which various UDP-activated sugar precursors required for CPS synthesis are derived (18, 23). We therefore examined the transcription of *spd_0088* and Leloir pathway genes *galR* (transcriptional regulator), *galK* (galactokinase), and *galT* (galactose-1-phosphate uridyltransferase), as well as *lacD* (tagatose 1,6-diphosphate aldolase, the last enzyme of the tagatose-6-phosphate pathway), in D39 and D39Δ*luxS* grown in a fully synthetic chemically defined medium (CDM) (24) with Gal as the sole carbon source (CDM-Gal) with or without 10 μM AI-2. Transcription of *cps2A*, the first gene of the locus encoding type 2 CPS biosynthesis, was also assessed. No differences in *cps2A* transcription between D39 and D39Δ*luxS* were seen, with or without AI-2 in CDM-Gal, consistent with the previous RNA-seq data (result not shown). However, the expression of *galR*, *galK*, and *galT* was significantly lower in D39Δ*luxS* than in D39. Moreover, addition of AI-2 significantly increased the expression of all three genes in both strains. Indeed, exogenous AI-2 increased the expression of the Leloir pathway genes in D39Δ*luxS* such that it was essentially identical to that in D39 without added AI-2, thereby fully complementing the *luxS* mutation (Fig. 7B to D). Expression of the Gal ABC transporter *spd_0088* gene showed a trend identical to that of the Leloir pathway genes, but the differences did not reach statistical significance (Fig. 7A). However, the tagatose pathway gene *lacD* did not respond to exogenous AI-2 in either strain (Fig. 7E). Interestingly, unlike the findings in medium without added sugars (Fig. 5), in CDM-Gal, expression of *fruA* by D39 and D39Δ*luxS* in response to AI-2 mirrored that of the Leloir pathway genes (Fig. 7F). Importantly, in D39Δ*fruA*, AI-2 had no effect whatsoever on the expression of Leloir pathway genes *galR*, *galK*, or *galT* or of *spd_0088* (Fig. 7A to D). Thus, FruA is essential for the transcriptional response of galactose uptake and utilization genes to exogenous AI-2 that results in upregulation of CPS production and increased virulence. It is important to note that FruA itself plays no known catalytic role in Gal uptake or catabolism or CPS synthesis. Thus, our findings that the baseline Leloir pathway gene expression, total CPS production, and virulence of D39Δ*fruA* are similar to or even higher than those of D39 do not conflict with the hypothesis that FruA transduces the AI-2 signal.

**FIG 8:**
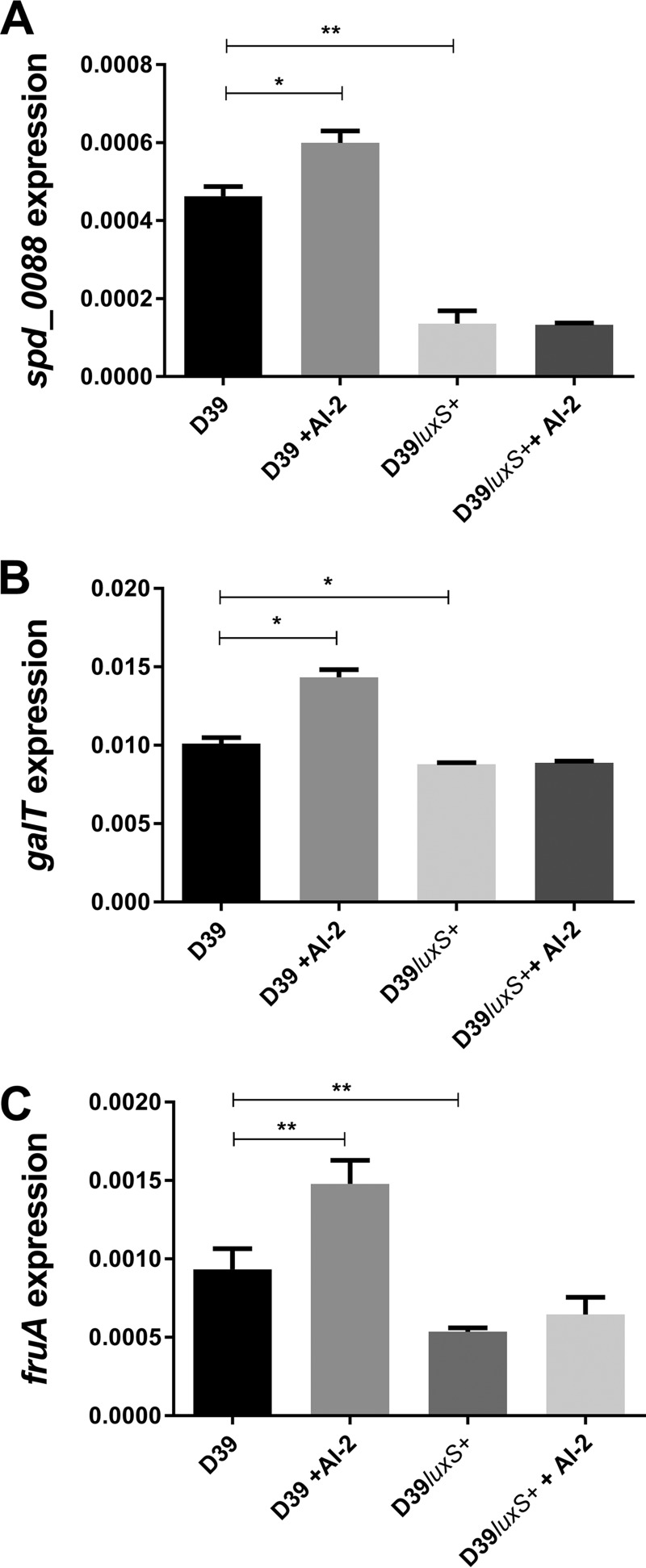
Relative expression of *spd_0088* (A), *galT* (B), and *fruA* (C) by D39 and D39*luxS*^+^ grown in CDM-Gal with or without 10 μM AI-2 was quantitated by qRT-PCR with 16S rRNA as an internal standard. Data are the total expression relative to 16S rRNA (mean ± the standard deviation of three independent experiments). *, *P* < 0.05; **, *P* < 0.01 (Student’s unpaired *t* test).

**FIG 9:**
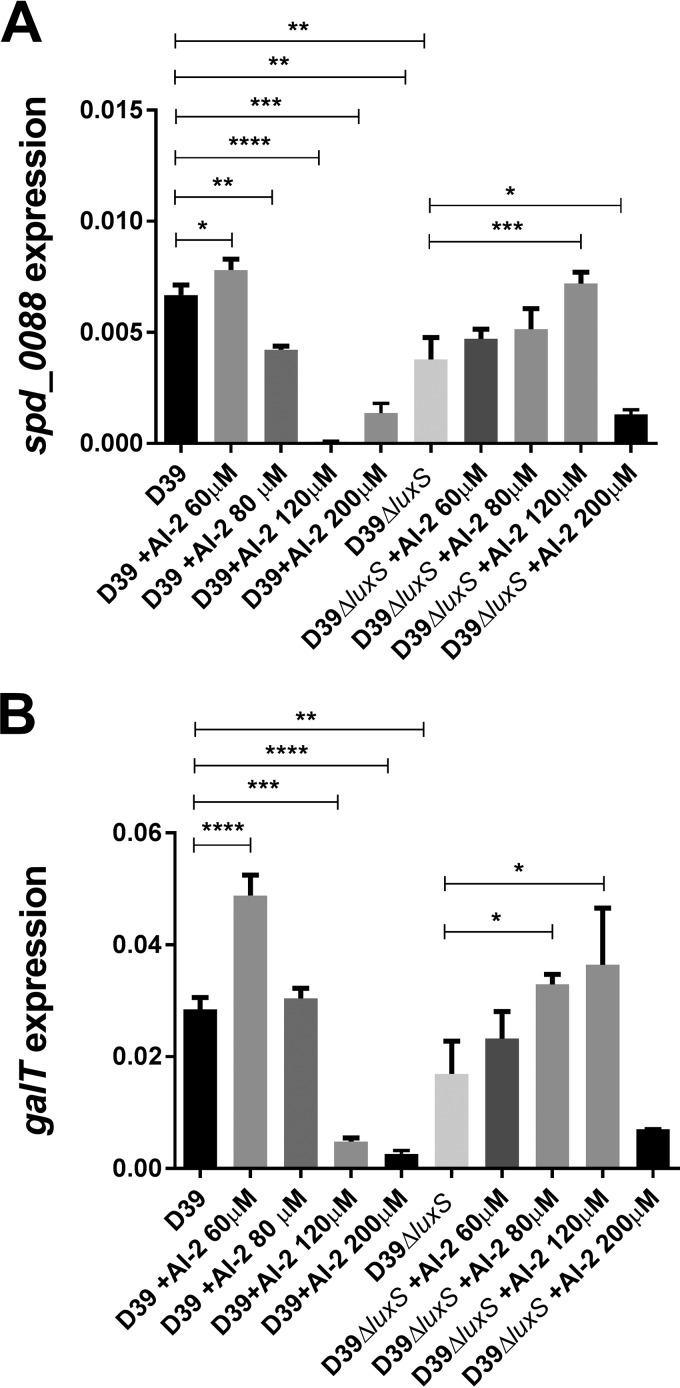
Relative expression of *spd_0088* (A) and *fruA* (B) by D39 and D39Δ*luxS* grown in CDM-Gal. D39 and D39Δ*luxS* were treated with 0, 60, 80, 120, or 200 μM AI-2, and transcripts were quantitated by qRT-PCR with 16S rRNA as an internal standard. Data are total expression relative to 16S rRNA (mean ± the standard deviation from three independent experiments). *, *P* < 0.05; **, *P* < 0.01; ***, *P* < 0.001; ****, *P* < 0.0001 (Student’s unpaired *t* test).

In order to account for the lower CPS production and avirulence of the *luxS*-overexpressing strain observed in Fig. 2 and 4, we then examined *spd_0088*, *galT*, and *fruA* expression in D39*luxS*^+^ versus D39 (Fig. 8). In D39, all three genes were significantly upregulated in the presence of exogenous AI-2. However, all three genes were significantly downregulated in D39*luxS*^+^ relative to D39 and expression did not increase in the presence of AI-2. These findings further illustrate the tight nexus between galactose uptake/catabolism and CPS production. These data also indicate that there is an optimum AI-2 concentration range above which responses to the QS molecule are switched off. This was further examined by measuring *spd_0088* and *galT* expression in D39 and D39Δ*luxS* exposed to higher AI-2 concentrations. In D39, addition of 60 μM AI-2 upregulated *spd_0088* and *galT*, but these genes were significantly downregulated at 80 and 120 μM AI-2, respectively (Fig. 9). In D39Δ*luxS*, exogenous AI-2 remained stimulatory at concentrations of up to 120 μM, but significant downregulation occurred at 200 μM. This higher inhibitory threshold is presumably attributable to the absence of baseline endogenous AI-2 synthesis in the *luxS* mutant.

## DISCUSSION

AI-2 released by LuxS activity has been recognized as a universal language for bacterial communication (25). However, there has been uncertainty as to whether the LuxS/AI-2 system has a true QS function in Gram-positive bacteria or just a metabolic role as a component of the activated methyl cycle. Lack of any knowledge of the AI-2 receptor in Gram-positive bacteria has also hindered investigations of how AI-2 signals are transduced in these organisms. In *S. pneumoniae*, the effects of *luxS* deletion include reduced biofilm formation and virulence, as well as effects on genetic competence and fratricide (16, 19, 26). Thus, *luxS* appears to lie at the center of a complex QS regulatory network (11, 27, 28). Recent availability of synthetic DPD, which spontaneously cyclizes to form biologically active AI-2 (29), has enabled experimental complementation of *luxS* mutant phenotypes. In the present study, we have used synthetic AI-2 to complement defects in pneumococcal growth and CPS production *in vitro* in media containing Gal, as well as defects in virulence seen in D39Δ*luxS* in a murine i.n. challenge model. These findings confirm that LuxS/AI-2 is a functional QS system in pneumococci.

Our findings also highlight the importance of Gal utilization by pneumococci *in vivo*. This sugar is the dominant carbon source available to pneumococci in the upper respiratory tract, where Glc is in very low abundance (30, 31). This Gal is largely derived from host glycoconjugates by the sequential action of NanA (neuraminidase) and Bga (β-galactosidase) (32, 33). Although D39Δ*luxS* grew normally in C+Y-Glc, there was a major growth defect in C+Y-Gal that was complemented with exogenous AI-2. These findings paralleled *in vivo* bacterial loads in the murine model, where the capacity to colonize the nasopharynx and spread to deeper tissues was attenuated in D39Δ*luxS* unless exogenous AI-2 was provided. Further *in vitro* studies showed that *luxS* mutation diminished resistance to phagocytosis by macrophages, while addition of AI-2 enhanced such resistance in both D39 and D39Δ*luxS*. Effects of *luxS* mutation and AI-2 treatment on the above *in vitro* and *in vivo* parameters were entirely consistent with differences in total CPS production in C+Y-Gal.

Despite the above observations, transcriptomic analyses showed no significant differences in the expression of any of the genes in the CPS biosynthesis locus, nor were there any differences in *cps2A* transcription, as judged by qRT-PCR. However, our data show that AI-2-mediated effects on CPS production could be driven by the availability of activated sugar precursors for CPS biosynthesis. Exogenous AI-2 upregulated genes encoding the Gal ABC transporter Spd_0088 and enzymes of the Leloir pathway (GalR, GalK, and GalT) in both D39 and D39Δ*luxS*, and the expression of all of these genes was lower in D39Δ*luxS* than in D39. When Gal is the only available sugar, as predominantly occurs in the respiratory tract, this is the only pathway capable of generating G1P, from which various UDP-activated sugar precursors required for CPS synthesis are derived (18, 23). The only other known Gal uptake system in *S. pneumoniae* is a PTS-like system that imports Gal as Gal-6-P. This can feed into the glycolytic pathway via the tagatose-6-P pathway, but it cannot be used to directly generate CPS precursors (23).

Since it has been shown that AI-2 acts as a QS signaling molecule that upregulates CPS production and virulence in *S. pneumoniae*, this then leads to the logical question of how the signal itself is transduced. In Gram-negative bacteria such as *E. coli* and *Salmonella* spp., AI-2 is imported by the Lsr transport system, which is repressed in the absence of an AI-2 signal by LsrR. When AI-2 is present, it is phosphorylated by LsrK, which relieves LsrR repression, activating the Lsr operon, and massively amplifying AI-2 internalization via the LsrABCD permease complex (34). It has been suggested that the purpose of this is to deplete the external environment of AI-2, thereby denying other bacteria in the niche, e.g., the gut, of a QS signal. However, the actual AI-2-mediated QS event that sets this cascade in motion involves a PTS that delivers AI-2 to LsrK. Mutation of *ptsI*, which encodes the enzyme I component common to multiple PTS uptake systems, prevents internalization of AI-2 and its phosphorylation by LsrK. However, the identity of the individual PTS permease that internalizes AI-2 remains unknown, and no single mutation in any of the known PTS permeases is capable of blocking Lsr-mediated uptake completely (34).

Gram-positive bacteria lack any homologues of either the Lsr permease or the LuxP/LuxQ phosphorelay system that internalizes AI-2 in *Vibrio* spp. (35). However, in the present study, we have shown unequivocally that the fructose PTS component IIABC FruA is necessary for AI-2 signaling in *S. pneumoniae*. D39Δ*fruA* cannot respond to exogenous AI-2, as indicated by lack of induction of *spd_0088*, *galR*, *galK*, or *galT*. Furthermore, there was no increase in total CPS production or virulence when D39Δ*fruA* was treated with AI-2 either *in vitro* or *in vivo*. FruA was identified as a candidate AI-2 receptor, as *fruA* was one of only four genes/operons whose expression level changed in both D39 and D39Δ*luxS* on exposure to AI-2, as judged by transcriptomic analyses. Mutation of two of the other genes (*piuA* and *adhE*) had no impact on AI-2 signaling, while mutation of the remaining AI-2-responsive gene (*brnQ*) had pleiotropic impacts that rendered the mutant incapable of growth in the CDM used in our transcriptional studies. Regulation of a sensor by its cognate ligand is an essential feature of a true QS system. In this context, it is interesting that expression of the *fruA* homologue in *Staphylococcus epidermidis* has also been shown to respond to exogenous AI-2 (36).

Here, we propose a model of the modulation of pneumococcal virulence by AI-2 whereby pneumococci establishing colonization of the human nasopharynx may initially be present at a low density but are able to sense AI-2 in the external environment released by themselves and by other commensal nasopharyngeal microorganisms. Recognition and import of AI-2 by the FruA PTS would both internalize AI-2 and perhaps phosphorylate it to form AI-2-P, which then would no longer be able to cross the membrane, trapping it within the cell. We attempted to detect AI-2-P by ^2^H nuclear magnetic resonance analysis of D39 and D39Δ*luxS* cell extracts before and after exposure to exogenous AI-2 but were not successful. Nevertheless, our data clearly show that AI-2 upregulates the Gal ABC transporter and the Leloir pathway enzymes, which are under the control of GalR, which is also upregulated by AI-2. It is tempting to speculate that AI-2-P is capable of phosphorylating GalR, thereby directly activating (or relieving repression of) the *gal* operon. In *S. pneumoniae*, GalR is known to be phosphorylated at residues S317, T319, and T323 (37). Thus, mutational analysis of these GalR residues in D39 may be informative. Regardless of the precise molecular mechanism, the above events would confer the capacity to import free Gal into the respiratory tract, enabling its utilization as a carbon source in general and for synthesis of CPS precursors in particular. Increased CPS production would, in turn, enhance resistance to phagocytosis and increase the capacity of the pneumococcus to invade deeper tissues.

Our findings provide strong evidence implicating FruA as a receptor/transporter for AI-2 in *S. pneumoniae*. While further studies are required, FruA is unequivocally essential for transduction of AI-2-mediated signaling. FruA is highly conserved among Gram-positive bacteria and may therefore represent a target for novel antimicrobials that would attenuate colonization and interfere with disease progression. Such agents would not be expected to be bactericidal and so may have a lesser impact on the microbial ecology of the host and also not apply selective pressure for evolution of resistance.

## MATERIALS AND METHODS

### Bacterial strains and growth conditions.

The *S. pneumoniae* strains used in this study are listed in Table 2. Strains were routinely cultured on Columbia blood agar base supplemented with 5% horse blood at 37°C in 95% air–5% CO_2_. Blood agar plates were supplemented with 0.2 μg/ml erythromycin or 200 μg/ml spectinomycin for the selection of mutant strains. Growth experiments with pneumococci in liquid culture were performed with a casein-based semisynthetic medium (C+Y) (21). For animal challenge experiments, pneumococci were grown in nutrient broth supplemented with 10% (vol/vol) horse serum. Gene expression experiments were performed with a CDM (38) supplemented with catalase (10 U/ml; Sigma) at 37°C under static conditions. The AI-2 precursor molecule, DPD, was purchased from Omm Scientific Inc., TX.

**TABLE 2:** *S. pneumoniae* strains used in this study

Strain	Description	Source or reference
D39	Capsular serotype 2	NCTC 7466
D39Δ*luxS*	*luxS* deletion-replacement mutant (Spec^r^)	This study
D39Δ*fruA*	*fruA* deletion-replacement mutant (Spec^r^)	This study
D39Δ*adhE*	*adhE* deletion-replacement mutant (Spec^r^)	This study
D39*luxS*^+^	D39 carrying pALT2::*luxS*	16
D39Δ*piuA*	*piuA* deletion-replacement mutant (Ery^r^)	47

### Construction of mutant strains.

Genes were deleted from *S. pneumoniae* D39 and replaced with an erythromycin or spectinomycin resistance cassette by transformation with a linear DNA fragment constructed by overlap extension PCR (39) with primers listed in Table 3.

**TABLE 3:** Primers used in this study

Primer	Sequence (5′–3′)
16S F	GGTGAGTAACGCGTAGGTAA
16S R	ACGATCCGAAAACCTTCTTC
galR-RT-F	TCTCTATCGCCGACCGTATCC
galR-RT-R	GGTGTAGCCCAGCTCTTCAG
galT-RT-F	GTGGGAGAAGGTGTTTTGGA
galT-RT-R	ACGCGCAGTCTGACTATCCT
galK-RT-F	CACGTTTCTCTGGAGCATGA
galK-RT-R	ATGGCACAGCCACTAAAACC
lacD-RT-F	CATCGGTTCTGAGTGTGTGG
lacD-RT-R	AAAGCGTGGGTCTGAAAAGA
SPD0088-RT-F	ATTCGCAACGTAACCATTCC
SPD0088-RT-R	AAGCTGACCAGCATTGTGTG
SPD0546-RT-F	CCATCGCAGGTTTTGTCTTT
SPD0546-RT-R	ACGTGGGGTAGCAAAGAATG
SPD1834-RT-F	TGCTCCTGAAAACTGTGTGC
SPD1834-RT-R	ACCTACCCCAAGAGCTGGTT
piuA-RT-F	AGGGCTCCTTCTCCATGATT
piuA-RT-R	GGGAGTAGGAAATCCCCAAA
SPD0546 For	GCCTACTATTTACAGGAGAA
SPD0546 Rev	AAAATCGCCGCACTGACCTTG
SPD0546 spec for	AAATAACAGATTGAAGAAGGTATAATGAAATGGAATAATCACTAAT
SPD0546 spec Rev	TATGTATTCATATATATCCTCCCTTTTTTAGCCATAAAAATCTCCT
luxS for	TGGACCAGCCCTAGCCTTTGAA
luxS Rev	CACACTTGACTAAGGAAGAC
luxS spec for	AAATAACAGATTGAAGAAGGTATAATCTCACACCACCGTACGTA
luxS spec Rev	TATGTATTCATATATATCCTCCTCGTTGCTCCTGAGACAGA
adhE for	GAGATTCCTTATGACAAGAA
adhE Rev	TACTAGCTTATTATTCTAG
adhE spec for	AAATAACAGATTGAAGAAGGTATAATGTTTATCAGTCTAGAAG
adhE spec Rev	ACAAAGGATATCGTTCCTGAGGAGGATATATATGAATACATA
J253	GAGGAGGATATATTTGAATACATACG
J254	TTATAATTTTTTTAATCTGTTATTTAAATAGTTTATAGTTA
cps2A-RT-F	TGTCAGCTCTGTGTCGCTCT
cps2A-RT-R	TATCAGTCCCAGTCGGTGCT

### Growth assays.

Analysis of pneumococcal growth was performed in 96-well microtiter plates (200-μl culture volume) at 37°C. Culture optical density at 600 nm (OD_600_) was measured at 30-min intervals over a 24-h time period with a Spectromax spectrophotometer (Molecular Devices).

### Murine infection model.

Our animal experiments were approved by the University of Adelaide Animal Ethics Committee. Female outbred 4- to 6-week-old CD-1 (Swiss) mice were anesthetized and inoculated i.n. with 10^7^ CFU of wild-type D39, D39Δ*luxS*, or D39*luxS*^+^ in 50 µl of medium as described previously (40). Two groups of five mice were used for each strain tested, and at time zero and 6, 12, and 18 h postinfection, one group received 1 μg of AI-2 in 10 µl of PBS i.n. (without anesthesia), while the other group received 10 μl of PBS as a control. Nasal wash, nasopharyngeal tissue, lung, and blood samples were collected and processed as previously described at 24 h postinfection. Samples were homogenized where appropriate, serially diluted, and plated onto blood agar for enumeration of viable pneumococci as previously described (40, 41). Statistical analyses of log-transformed data were performed with a two-tailed Student *t* test; a *P* value of <0.05 was defined as statistically significant.

### Histological examination of lung tissue.

Twelve hours after an i.n. challenge or no challenge (control), lungs were removed from euthanized mice, fixed in 4% formaldehyde overnight at 4°C, embedded in paraffin, sectioned, stained with H&E, and examined by light microscopy (Axiophot; Zeiss, Germany). Digital images were captured and processed with a CCIZ soft imaging system and analySIS LS Research software. Lung pathological scores (maximum = 8) for each mouse were determined by a blinded observer according the following scheme: congested capillaries, scored 1 to 2; thickened alveolar wall, scored 1 to 2; secretions and hemorrhage in alveolar and bronchiolar spaces, scored 1 to 2; parenchymal hemorrhage and neutrophil infiltration, scored 1 to 2.

### Culture and differentiation of THP-1 cells and phagocytic assay.

All tissue culture media and reagents were obtained from Gibco. THP-1 cells (ATCC TIB-202) were grown in 95% air–5% CO_2_ at 37°C in complete RPMI medium (RPMI with phenol red, supplemented with 10% fetal calf serum, 10 mM HEPES, 50 IU/ml penicillin, and 50 µg/ml streptomycin). Ten-centimeter dishes were seeded with 3.5 × 10^6^ THP-1 cells and differentiated by adding phorbol 12-myristate 13-acetate to a final concentration of 10 ng/ml and incubating them for 3 days at 37°C in 95% air–5% CO_2_. Differentiated cells attach to the plastic surface. Following the 3 days of incubation, cells were washed twice with complete RPMI and then rested at 37°C in 95% air–5% CO_2_ in complete RPMI for a further 3 days. At 90% confluence, differentiated THP-1 cells were incubated with bacteria (100 µl) at a 10:1 ratio of bacteria to THP1 cells. After 45 min, plates were washed and cells were reincubated with 10 μg/ml penicillin and 200 μg/ml gentamicin (Sigma, Germany) for 30 min to kill extracellular pneumococci. Intracellular bacteria were enumerated after lysis with 0.01% Triton X-100 by plating serial dilutions on blood agar.

### Uronic acid assay.

Pneumococcal CPS samples were prepared as previously described (42). In brief, deoxycholate-lysed pneumococci were treated by adding 100 U of mutanolysin (Sigma, Australia), 50 U of DNase I (Roche, Australia), and 50 μg of RNase A, incubating them overnight at 37°C, and treating them with 100 μg/ml proteinase K at 56°C for 4 h. Uronic acid was then quantitated colorimetrically as described previously (42).

### Bacterial RNA extraction and RNA-seq analysis.

*S. pneumoniae* strains were grown in C+Y without added sugars to an OD_600_ of 0.15 and then treated with or without 10 μM AI-2 for 30 min. RNA extraction was performed with a Qiagen RNase minikit according the manufacturer’s protocol. Each sample was derived from three independent cultures (biological replicates) and pooled prior to transcriptome sequencing (RNA-seq) analysis. Total RNA was submitted to the Australian Genome Research Facility (Melbourne, Australia) for sequencing. Briefly, the Epicentre Bacterial Ribozero kit (Illumina) was used to reduce the rRNA content, and then the Ultra Directional RNA kit (New England Biolabs) was used to generate bar-coded libraries. Prepared libraries were then sequenced with the Illumina HiSeq 2500. Trimmed RNA-seq fastq files were then mapped to the D39 reference genome with BOWTIE2 version 2.2.3 (43). Counts for each gene were obtained with the aid of BEDTools (44), and differential gene expression was examined with DESeq (45).

### Real-time qRT-PCR.

For qRT-PCR analyses, cells were grown in CDM-Gal to an OD_600_ of 0.2 and treated with or without 10 μM AI-2 for 30 min. RNA was extracted from triplicate cultures as described above, but individual rather than pooled extracts were analyzed. Gene expression was assayed by one-step relative real-time qRT-PCR in a Roche LC480 real-time cycler essentially as described previously (40). The specific primers used for the various genes are listed in Table 3, and they were used at a final concentration of 200 nM. As an internal control, primers specific for 16S rRNA were employed. Amplification data were analyzed by the comparative critical threshold (2^−ΔΔ*CT*^) method (46) and are presented as total expression relative to that of the 16S rRNA gene.
